# “Pressure” Anesthesia

**Published:** 1907-05-15

**Authors:** Herman Prinz


### "PRESSURE" ANESTHESIA.

BY HERMAN PRINZ, M.D., D.D.S.

By pressure anesthesia, pressure cataphoresis, peridental anesthesia or contact anesthesia as this process is variously termed, we understand the introduction of a local anesthetizing agent in solution by mechanical means through the dentine into the pulp for the purpose of rendering this later organ insensible to pain. Simple hand pressure with a suitable instrument, the hypodermic syringe or the so-called high pressure syringe, is recommended for such purposes. Before describing the *modus operandi* of the various methods, the histological structure of the dentine should be briefly recalled. Dentine is made up of about 72 per cent of inorganic salts, about ten per cent of water and an inorganic matrix constituting the remaining percentage. The dentine is perforated by a large number of tubules, radiating from the pulp cavity, more or less wave-like, towards the periphery, where they branch off, forming a deldoid network. The Dentinal tubules are filled with the processes of the odontoblasts and are known as Tomes' fibres. The odontoblasts form a continuous cover over the pulp. The dentinal fibrils are protoplasmic in their nature; normally they do not carry sensation in the sense of the word as we understand this term as attributed to a nerve fiber; we can cut, file or otherwise injure the sound dentine without the slightest inconvenience to the patient. When the fibers have become highly irritated, the mere touch may at once produce a paroxysm of pain.

Regarding the principle of "pressure" anesthesia, the literature of the last five years is filled with speculative statements. It is generally believed that a liquid anesthetic can be forced through the normal dentine of a healthy tooth. Some writers have even stated that they were able to demonstrate under the microscope the pressure of cocain in a pulp removed from a tooth treated by pressure anesthesia. For plausible reasons such explanations are not in accordance with the physical and physiological laws governing this process. From a physical point of view, it should be remembered that water confined in a closed tube can only be compressed very little (about 1-2500 of an inch in a tube 40 inches long), nor can two bodies occupy the same space at the same time. This same law holds good in regard to the dentinal tubules. If a cocain solution is held in close contact with the protoplasmic fibers, the absorption of cocain takes place in accordance with the law of osmosis. The imbibition of the anesthetic is enhanced by employing a physiological salt solution as a vehicle. On the other hand, living protoplasm reacts unfavorably against the ready absorption of substances by osmosis, for two reasons: First, its albumin molecule is relatively large and not easily diffusible; and second, as an integral part of its life it possesses "vital" resistance (*vis a tergo*) toward foreign bodies. These factors are sufficiently demonstrated by the fact that it is almost impossible to stain living tissue. Dehydration of the protoplasm increases the endosmosis of the anesthetic solution markedly. According to Hertwig, the protoplasm of the cell possesses a twofold physiological function: It may transfer irritating impulses from one cell to another, and it may transport materials which have been absorbed.

When we apply the same "pressure" anesthesia upon carious dentine the above statements do not hold good. We are able to press fluids quite readily through carious dentine we must bear in mind that such dentine has been largely deprived of its inorganic salts, leaving an elastic, spongy matrix in position. By drying out this dentine and then confining the anesthetic solution under a suitable water-tight cover, the pressure applied by the finger is quite sufficient to obtain the desired results. Colored fluids, viz., aqueous eosine solution, may be readily pressed through such dentine, and even stain the pulp. From the physiologist's view-point, Preyer explains the theory of local anesthesia as follows: Cocain and similar chemicals possess a definite affinity for living protoplasm of the nerve cell; they enter with it into a labile union. This union is the factor which produces local anesthesia, and the anesthesia lasts until this temporary union is broken up by releasing the chemical—not as the original cocain, however, but as an inert compound of a simpler structure. In other words, the tissues will rid themselves of the poison in some unknown manner.

From the foregoing it will readily be observed that the so-called high-pressure syringes do not possess any merit whatsoever relative to "pressure" anesthesia. It is claimed that 3,000 pounds of pressure may be obtained with some of these instruments. This may be true but it is equally true that such a syringe filled with an eosine solution and connected with a sound tooth by cementing it into a drill hole will not force the liquid into the tooth, even if the pressure is increased to five atmospheres. We have not tried to register the amount of pressure which is necessary to burst a tooth. The pressure which can be produced by a good-working all-metal syringe, holding it between the index and middle fingers and forcing the piston with the thumb, amounts to 250 to 300 pounds with the average man. The pressure required in "pressure"

anesthesia to produce a perfect contact is much less than the above force, consequently any strongly built syringe answers the purpose. The all metal platinoid syringe of the Manhattan Dental Company has given good results in all our gauge tests and in clinical work.

### METHODS OF ANESTHETIZING THE PULP.

I. The pulp is wholly or partially exposed: Isolate the tooth with the rubber dam and clean it with water and alcohol. Excavate the cavity as much as possible, and if the pulp is not exposed, dehydrate with alcohol and hot air. Saturate a pledget of cotton or a piece of spunk with a cocain solution, place it into the prepared cavity and cover it with a piece of vulcanizable rubber, and with a suitable burnisher apply slowly increasing, continuous pressure from one to three minutes. The pulp may now be exposed and tested. If it is still sensitive, repeat the process. Loeffler states that "This pressure may be applied by taking a short piece of orange wood, fit it into the cavity as prepared and direct the patient to bite upon this with increasing force. In this way we can obtain a well directed, regulated force or pressure, and with less discomfort to the patient and operator." Miller describes this process as follows: "After excavating the cavity as far as convenient and smoothing the borders of it, take an impression in modeling compound, endeavoring to get the margins of the cavity fairly well brought out; put a few threads of cotton into the cavity and saturate them thoroughly with a five to ten per cent solution of cocain; cover this with a small bit of rubber dam and then press the compound impression down upon it. We obtain thereby a perfect closure of the margin, so that the liquid cannot escape, and one can then exert pressure with the thumb sufficient to press the solution into the dentine."

II. The pulp is covered with a thick layer of healthy dentine: With a very small spade drill bore through the enamel or direct into the dentine at a most convenient place, guiding the drill in the direction of the pulp chamber. Blow out the chips, dehydrate with alcohol and hot air and apply the syringe provided with a special needle, making as nearly as possible a water tight joint. Apply slowly, continuous pressure for two to three minutes. With a bur the pulp should now be exposed, and if still found sensitive the process is to be repeated.

Recently a method has come into vogue which allows successful anesthetization of the pulp by injecting the anesthetic solution around the apex of the tooth. The spongy alveolar process, which contains lymph channels, allows the ready penetration of the fluid. The injection should be made close to the bone. pushing the needle slowly toward the apex while the fluid is deposited drop by drop. No wheal should be raised by the injection; otherwise the benefits of the pressure from the dense gum tissue are lost.

As stated above, the protoplasm of the cell primarily transfers irritaton, and secondly, transmits absorbed materials. Therefore the anesthetic solution has to pass through the entire dentinal fibre before the nerve tissue of the pulp proper is reached. Consequently a certain period of time is required before the physiological effect of the anesthetic is manifested. This latent period is dependant upon the thickness of the intermediate layer of dentine. The successful anesthetization of the pulp depends largely upon this most important factor of allowing sufficient time for the proper migration and action of the drug.

Since the introduction of the cocain-adrenalin combination for the purpose of mitigating pain in operative dentistry, local anesthesia has achieved results which, considering its safety and reliability, cannot be obtained with cocain or any of its substitutes. From our investigations into the nature of the above combination, we are convinced that it is the safest and most effective means of producing local anesthesia upon a rational basis so far known.—*Dental Era.*

